# Amber “Alchemy”: Recreating and Investigating Yellow Glass Formulations

**DOI:** 10.3390/ma17235699

**Published:** 2024-11-21

**Authors:** Catarina Reis Santos, Andreia Ruivo, Ana Carneiro, João Pedro Veiga, Teresa Palomar, Inês Coutinho

**Affiliations:** 1 Department of Conservation and Restoration, NOVA School of Science & Technology, Campus Caparica, 2829-516 Caparica, Portugal; a.ruivo@fct.unl.pt (A.R.); jpv@fct.unl.pt (J.P.V.); 2 VICARTE Research Unit, NOVA School of Science & Technology, Campus Caparica, 2829-516 Caparica, Portugal; t.palomar@csic.es; 3 CIUHCT Research Unit, NOVA School of Science & Technology, Campus Caparica, 2829-516 Caparica, Portugal; anacarneiro613@gmail.com; 4 CENIMAT/I3N—Centro de Investigação de Materiais, NOVA School of Science & Technology, Campus Caparica, 2829-516 Caparica, Portugal; 5 CSIC—Institute of Ceramic and Glass, Campus de Cantoblanco, 28049 Madrid, Spain

**Keywords:** glass recipes, amber, Portuguese arcana, 18th–20th century

## Abstract

Amber glass has been produced since at least the 6th century BC. Its value derives from its ability to mimic natural amber and, later, from its ultraviolet filtering properties. Until the 19th century, amber glass was widely used for the storage of food and medicines because its protective properties had been empirically recognized. This study investigates historical methods of amber glass production by using glass recipes from four Portuguese arcana (1793–1975) and focusing on Fe-S and Fe-Mn chromophores. Five recipes were reproduced under controlled laboratory conditions, resulting in 21 experiments. Of these, only 10 produced amber glasses were with different shades. Chemical compositions were analysed by WDXRF, while DSC and dilatometry were used to assess thermal properties. Vickers hardness tests and UV–visible absorption spectroscopy provided insight into mechanical strength and chromophore presence. The study found that FeS amber glass was more difficult to produce than Fe-Mn amber glass, given the complex variables involved in the former, such as SO_3_ volatility affecting the final product. Reproduction of historical recipes showed that, even without modern chemical knowledge, historical glassmakers developed practical, empirical methods for making amber glass. These findings contribute to a broader understanding of glass conservation and highlight the importance of historical glass recipes for the interpretation and conservation of glass objects.

## 1. Introduction

Historically, amber glass has been produced and traded by humans since at least the 6th century BC [1]. Its synthesis initially served to create beads that imitated the natural and valuable amber (a fossilized resin). By the Roman era, glassmakers had mastered the production of this colour through meticulous control of their raw materials. Amber glass, a term used to describe hues ranging from golden yellow to dark brown, became popular for crafting vessels around the 4th–3rd century BCE [2,3]. Around the 19th century, it was realised that this glass had specific properties—nowadays understood as a capacity to filter the wavelength ultraviolet and visible radiations—contributing to preserve its content (e.g., food and medicines) [2]. In fact, by 2006, it was estimated that amber glass constituted 50% of all glass containers in the USA, predominantly for beer packaging [4].

**Figure 1:**
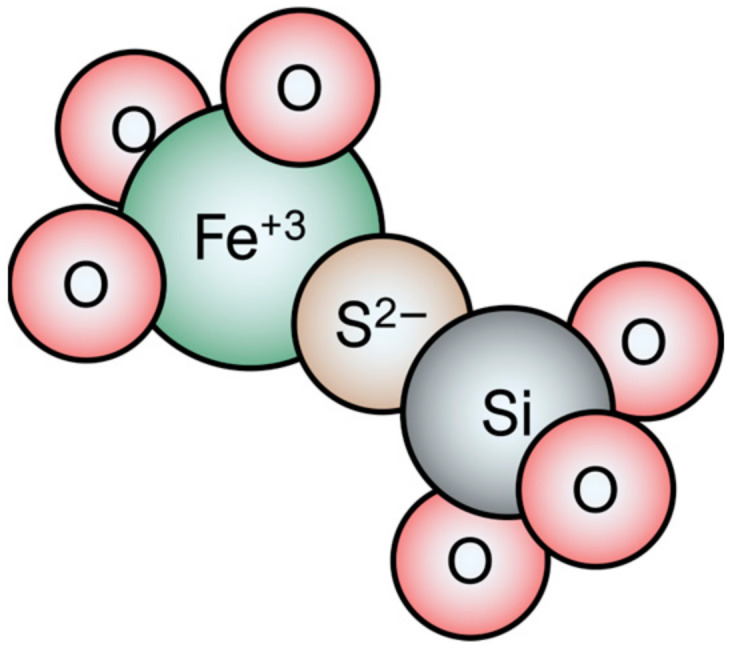
Illustration of the amber chromophore, adapted from Ross and Myers 2006.

In imparting colour to glass, iron is a versatile colourant in glass, producing colours from yellow (Fe^3+^) to blue (Fe^2+^), with green being the most common colour due to the combination of both oxidation states. However, the amber colour discussed here—a brownish yellow—differs from colours typically produced by iron alone. The amber chromophore, now understood to be a ferri–sulphide complex, was long attributed to carbon, a necessary ingredient for its production [2,4,5]. In 1959, Weyl provided a comprehensive historical review of “carbon-amber glasses”, exploring earlier theories, such as those proposed by Eckert and Zschacke in 1928, who suggested that the colour was associated with iron sulphide formation, although they lacked analytical proof [6]. It is notable that this glass colour was probably among one of the most difficult to attribute to a colouring agent. It was only in 1965 that Brown and Douglas, through a series of experiments that involved the production of several variations of amber glass recipes, proposed that sulphur and iron were probably involved in the amber colouration [5]. Current understanding posits that the amber chromophore is composed of an Fe^3+^ ion with three bridging oxygens (O^2−^) and one S^2−^ ion, as shown in Figure 1 [4].

Despite various advancements, producing amber glass has proven to be a challenging process. The recipe appears simple—requiring only silica (as a network former), sodium (as a flux), lime (as a stabilizer), iron (typically present in the silica source), sulphur (often from sodium sources like natron), and a reductive agent such as carbon—but it requires careful control of redox reactions. The amber colour is obtained when the co-existence of an oxidized-iron species (Fe^3+^) and a sulphur-reduced species (S^2−^) is achieved [2]. The success of Roman glassmakers in producing this colour was probably due to their strict control of raw materials, including a source of silica with sufficient iron oxide, a source of sodium rich in sulphur, such as natron, and a reducing agent such as charcoal [3].

The Industrial Revolution introduced more complex recipes and materials in amber glass production, as evidenced in batch books and patents. For example, the use of pyrite (FeS_2_) as an iron and sulphur source became widespread [4,7]. Although amber glass can be made with inexpensive raw materials, glass factories in the mid-20th century often opted for more expensive components, such as uranium and selenium, to achieve and control the amber colour [6]. By the 1940s, researchers such as Lawton, A.J. Holland, and W.E.S. Turner had conducted extensive studies on amber glass production, showing that even with identical batch formulas, factories struggled to replicate colours consistently [6].

An alternative method for producing amber glass involves adding manganese oxide to the mix. In this case, the redox process must still be controlled, and the colour results from the balance between iron and manganese oxides (Equation (1)) and the mix of colours [2]. Achieving consistent amber colour remains to this day difficult, even when using modern techniques such as the use of real-time sensors and oxygen probes, allowing for precise adjustments to the redox environment during production.

Equation (1)—Iron and manganese redox reaction

(1) $${Mn}^{4+(brown/black)}+{2Fe}^{2+(blue)}⇌{Mn}^{2+(yellow)}+{2Fe}^{3+(yellow)}$$

This article explores how modern amber glass manufacturing can benefit from a scientific approach to historical recipes found in batch books, such as the arcana. By analyzing the technologies and knowledge of past periods, this research investigates amber glass production and its unique properties, particularly its ability to filter ultraviolet and visible radiation, while offering insights into predicting long-term glass degradation. Focusing on the industrial and technological aspects of amber glass production, this study recovers historical recipes to better understand raw materials, production methods, and the economic networks of the time. By examining amber-colored glass recipes from four Portuguese *arcana* (18th–20th centuries), this research underscores the value of continued investigation into the Portuguese glass industry, with potential benefits for modern glass production both locally and globally.

### Contextualizing the Recipes: The Arcana

Batch books, often referred as arcana, are invaluable historical records that provide unique insights into the glassmaking practices of the past. The term arcana, which means “secrets” in Latin, reflects the confidential nature of the information they contain [8,9]. These notebooks are not traditional books or treatises, but practical guides used by glassmakers, especially composers, to document their recipes, techniques, and personal experiences. Serving as essential references for ensuring consistency in production, these arcana detail the proportions of raw materials, furnace conditions, and, in some cases, the origin of the ingredients. Often intended for personal use, they were usually difficult for others to interpret, functioning more as a testament to the skill and indispensability of the glassmaker who carried them from factory to factory.

The Marinha Grande Factory arcanum (MG) dates to 1793–1798 and consists of five notebooks, possibly authored by three different individuals given the handwriting variations. Like in former research, these five notebooks are to be studied as a whole [10,11,12]. Marinha Grande Factory, one of Portugal’s most famous glass factories, operated from 1719 to 1922, producing mainly crystal and window glass [13]. The Castro and Oliveira Guerra arcanum (COG), attributed to two specific glassmakers, is dated from 1875 to 1925, and is the only arcanum in Portugal to be both published and fully contextualized [8]. Meanwhile, the Gaivotas Factory (GF) arcanum (1935–1975) likely belonged to Francisco António Rodrigues, a glassmaker whose family later donated the book for study. This factory, founded in 1811 in Lisbon, was renowned for its Art Deco glassware and laboratory bottles [13]. The most recent discovery is the arcanum from the Northern Portuguese Glass Centre (NPGC), dating to 1955, recovered from Oliveira de Azeméis, a town where the first large-scale Portuguese glass factory was established in the 16th century [13].

Across these four arcana, a total of 69 yellow and amber glass recipes have been identified. The majority (67%) rely on the iron and sulphur complex as the chromophore, while 22% depend on a balance of iron and manganese. Uranium-based recipes account for 9%, and cadmium-based recipes make up 3%. Given this distribution, this study focuses on the amber-coloured glasses derived from the iron–sulphur complex and mixture of iron and manganese, excluding uranium and cadmium-based recipes.

Since achieving a proper balance between iron and sulphur represents a significant technological challenge, four recipes using this chromophore were selected for further investigation, along with one iron–manganese recipe for comparison. The study will assess the resulting colour and thermal and mechanical properties, such as glass transition temperature (Tg) and hardness. An additional goal is to determine if the glass produced aligns with the historical recipe descriptions, such as whether a “light amber” recipe indeed results in light amber glass.

This work seeks to validate the arcanum as a key historical source, not in theory, but also potentially tied to the creation of objects currently found in Portuguese museums. By examining the intricate processes of amber glass production in the Portuguese glass industry, this research contributes to a deeper understanding of the nation’s industrial heritage and its place in the global glassmaking history of the period.

## 2. Materials and Methods

### 2.1. Reproduction Methodology

**Figure 2:**
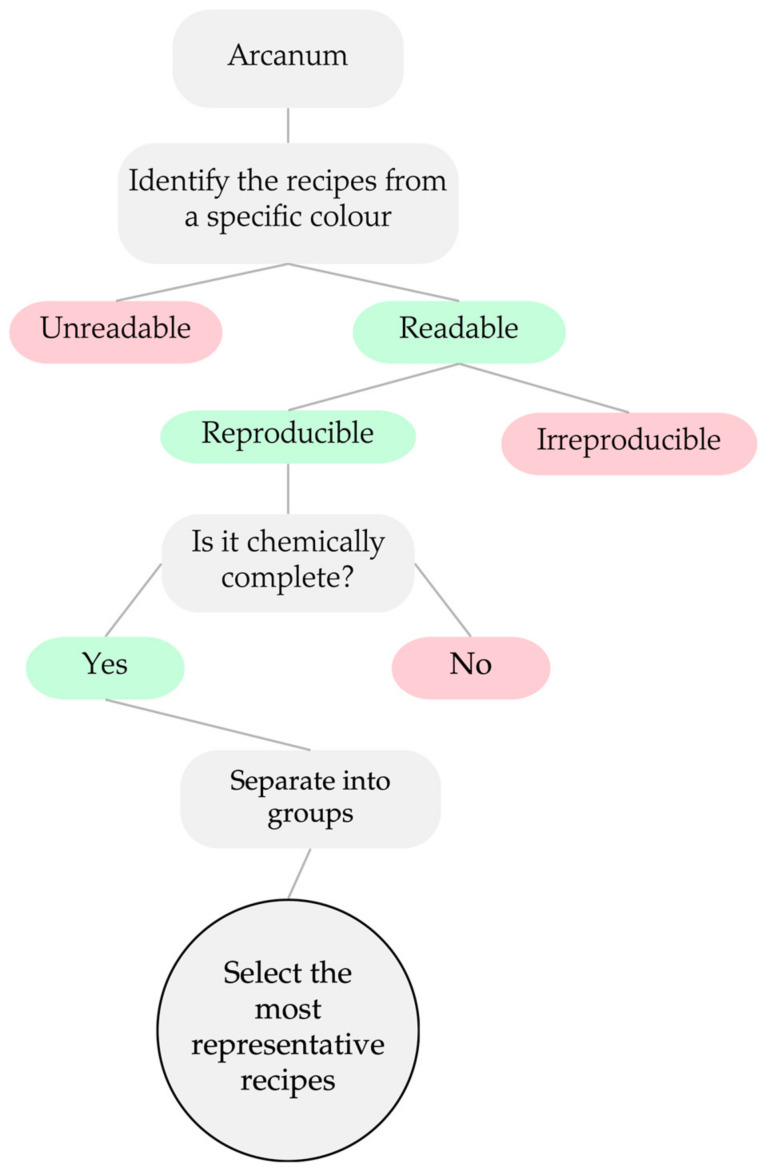
Decision tree of the process of interpreting and selecting the recipes to reproduce.

Before reproducing the glass recipes in the laboratory, it is essential to establish a replicable method for interpreting and selecting relevant recipes for reproduction (Figure 2). Initially, all glass recipes of a specific colour must be identified; in this case, the focus is on amber-coloured glass recipes. Subsequently, a thorough assessment should be conducted to ensure the legibility of these recipes, discarding any that are unreadable. Once only legible recipes are considered, the next step is to select those that appear to be reproducible.

In terms of reproducibility, it is crucial to ascertain the meaning of each ingredient, as the legibility of a recipe does not necessarily guarantee an understanding of its components. Furthermore, it is important to determine whether a historically accurate interpretation is feasible. For instance, if a recipe from a specific factory mentions sand as an ingredient, it is imperative to ascertain whether the sand has been previously analysed. Since iron is obtained from the sand source, it is vital to understand whether its iron content is known and whether there are historical objects from that factory that can be characterized to determine their iron content.

Finally, a check for chemical completeness should be conducted. Following the composition of any glass, these recipes should include a network former, a flux, and a stabilizer. Since this is a coloured glass, they should also incorporate the respective chromophore. Given that the formation of the FeS complex implies a reductive atmosphere during melting, it was also necessary to identify the organic compounds in the recipes.

In this work, the recipes from three of the four arcana presented above were studied, and the most representative of each has been selected. The most representative is the one that is similar to the majority of recipes of that colour from each arcana. It is important to note that while a selection of recipes was made, they are quite similar and share a key trait: the amount of iron, which is crucial for producing amber glass but is never specified, as it naturally occurs in the sand. Therefore, when a recipe failed to produce amber glass, variations were developed instead of altering the original formula. Table 1 shows the complete transcript of the selected recipes as they are written in the arcana (translated into English). Recipe NP3 belongs to the NPGC arcanum and is the recipe containing Fe-Mn. Three recipes were selected from the Gaivotas arcanum—G92, G130, and G192—each with different compositions and reducing agents. Finally, from the COG arcanum, only one recipe was selected, C98, featuring a simple composition similar to most of the recipes found some of the batch books in Corning’s Rakow Library

For the batch preparation, laboratory-grade reagents were used. As various studies have shown, the use of natural raw materials introduces many variables, complicating a historically accurate reproduction of glasses [14,15]. Factors such as the time of year when raw materials were collected (relevant in the case of vegetable sources), the preparation process before melting, and the initial characterization of these materials can pose challenges. In cases in which certain ingredients are known to be inherently impure, these impurities are simulated. For example, using sand as a silica source is always associated with iron oxide contamination. Reproducing a recipe from a specific batch book historically linked to a particular glass factory, using only pure silica dioxide, may not yield a historically accurate reproduction. To address this issue, and because iron oxide is essential to obtaining the amber-yellow colour, different approaches were employed to determine the amount of this oxide that should be added to the initial recipe for each arcanum. For the Castro and Oliveira Guerra arcanum recipes, the amount of Fe_2_O_3_ added was based on analyses conducted on the sands used by the Marinha Grande factory, as this arcanum is thought to have been used in that factory [16,17]. For the Gaivotas recipes, since there is no analysis or knowledge of the sands used, several historical colourless glasses produced at the factory were analysed to quantify the minimum amount of iron oxide required. For the NPGC, the approach was the same and the amount of iron oxide was also determined by analysing historical objects. The choice to use colourless objects to determine the percentage of iron in the sand came from the fact that they were the only historical objects we had associated with each of the factories. Considering that the sand would probably be the same for all glass compositions, the minimum iron value was determined.

Table 2 shows the amount of iron oxide used in each recipe, along with its variations. The reproduction process always starts with the original recipe, to verify its effectiveness without modifications. Next, several variables were individually modified, such as the amount of iron oxide, the addition of charcoal and the sulphur source. Given the complexity of the glass, recipe C98 was chosen to test all these variables due to its simpler composition, which facilitates the interpretation of results. In the Gaivotas arcanum, only the original interpretation of each selected recipe was tested.

The total amount of raw materials weighed for each recipe ranged from 50 to 60 g (Table 3), and each recipe mixture was homogenized by mixing it in a powder mixer (turbula) for at least 30 min. The mixture was then placed in the furnace at room temperature in an alumina crucible. This is one of the proofs that a large-scale reproduction is not exactly the same as a small-scale reproduction, and at this is one of the major problems in the study and reproduction of historical recipes.

Regarding the glass melting process, no recipes indicate information on the furnace or the melting temperature. Therefore, a temperature of 1450 °C has been chosen for melting, with a two hours’ dwell. This temperature may potentially exceed historical practices; however, it helps to optimise the time required to obtain an proper glass homogenization and, therefore, for each reproduction, making this study less time-consuming and more sustainable. When necessary, adjustments to the temperature and, consequently, the soaking time will be made, as the latter plays a crucial role in the success of glass production. It is important to note that the atmosphere inside the furnace was not controlled or measured. Previous studies have demonstrated that the atmosphere within the glass batch itself has more significant impact on the final result than the furnace atmosphere [18]. As for the procedure following the melting of the glass, some researchers left it in the furnace to cool, while others poured it onto a metal plate [14,15,19,20]. In previous work [9], the glass was left in the furnace to cool down to room temperature prior to testing; however, this caused difficulties in obtaining samples for analysis without cracks or fractures. Therefore, in this study, it was decided to pour the glass onto a metal plate and then subject the sample to an annealing process to remove any internal stresses, at approximately 470 °C for one hour, and to leave it to cool naturally in the furnace.

### 2.2. Characterisation of the Reproduced Samples

The obtained glass samples were characterised using the following techniques: wavelength dispersive X-ray fluorescence spectrometry (WDXRF), UV–vis absorbance spectroscopy, differential scanning calorimetry (DSC), dilatometry, the Vickers hardness test, colorimetry measurements and image capture with the stereomicroscope (SMZ Microscope with a colour camera Nikon DS-Fi3, Tokyo, Japan). The WDXRF was used to analyse the elemental chemical composition of the samples; UV–Vis spectroscopy to confirm the colourants imparting the amber colour; colorimetry was used to determine the colour coordinates and to compare tonalities between the FeS amber with the Mn-Fe amber recipes; and DSC and dilatometry were used to assess the workability of the glasses. Finally, the Vickers hardness test was used to test the possibility of cutting and engraving, or the hardness of the glass, in general.

The UV–Vis absorbance spectroscopy analyses were performed with Avantes AvaSpec-2048 fibber optic spectrometer (Avantes, Eebeek, The Netherlands), which operates at 200–1100 nm with a resolution of 2.4 nm. The emitted light was measured using a 200 μm reflection probe (Avantes FCR 7-UV-200), which consists of a central reading fibber, surrounded by six lighting fibers, each having a diameter of 200 μm. The spectra were obtained in absorbance mode, between 350 and 1050 nm, with an integration time of 13–18 ms and 20 scans.

The thermal analyses were performed using a Netzsch Pegasus^®^ DSC 404 F3 (Selb, Germany), which is equipped with a furnace that can reach temperatures up to 1550 °C. The experiments were conducted using a platinum crucible at a heating rate of 20 °C·min^−1^ in a nitrogen atmosphere. This technique provides the glass-transition temperature (Tg), and three analyses were carried out on each sample. In dilatometry, the glass transition temperature (Tg) was determined by dilatometry, employing a Netzsch Gerätebau dilatometer (model 402 PC) with a heating rate of 5 °C·min^−1^, and the equipment was operated by Cristina Ruiz Santa-Quiteria (Institute of Ceramic and Glass, Madrid, Spain).

The Vickers hardness of the glass samples was measured using Zwick-Roell Indentec test equipment (West Midlands, UK). Ten indentations were performed on each sample, with a measurement time of 10 s and a load of 0.5 kg.

Elemental analysis by WDXRF was conducted using a PANalytical XRF-WDS 4 kW AXIOS sequential spectrometer (PANalytical B.V., Almelo, The Netherlands) equipped with a Rh X-ray tube and four analyser crystals. The equipment was operated by Fernanda Carvalho (NOVA School of Science & Technology, Caparica, Portugal). The measurements were carried out under a helium flow in scanning mode to detect the maximum number of elements in the sample (Z > 8). Spectral deconvolution was performed using the iterative least squares method, and elemental quantification was based on the fundamental parameter approach, utilizing 15 certified secondary standards. This was achieved using the SuperQ software package and PANalytical’s standardless analytical program IQplus ((v.5.3A, PANalytical B.V., Almelo, The Netherlands). This methodology enabled a multi-element analysis of samples with varying quantities and complex chemical compositions in a relatively fast and straightforward manner. Due to the high resolution of the technique, spectral interferences, such as overlaps of characteristic X-rays from different elements, were minimal, allowing detection of elements even at trace levels (ppm).

Three colorimetric measurements of each sample were conducted with a Lovibond^®^ TR520 handheld spectrophotometer, (Dortmund, Germany) configured with an optical geometry of 8° viewing angle, diffused illumination, and a measurement aperture of 8 mm. Colour coordinates were calculated using CIE Illuminant D65, and are presented in accordance with the CIE L*a*b* system.

## 3. Results and Discussion

### 3.1. Recipe Identification

It should be noted that all four arcana are handwritten. In some cases, the calligraphy prevents a correct interpretation or identification of the written ingredients. However, the discussion around the reproducibility of a recipe is far more complex than the one around the calligraphy. The reproducibility is indelibly associated with the secrecy surrounding the arcanum (Figure 2). Sometimes these notebooks were written with code words and hidden information, but it also turns out that this non-reproducibility can be a consequence of the fact that the authors are so familiar with the subject that writing something so obvious leads them to often resort to abbreviations or expressions, which were then current or were part of the factory jargon, but no one knows their meaning nowadays. It is also important to note that most recipes are entitled “yellow glass”. However, various colouring agents can be included in this designation, such as uranium, cadmium, iron–sulphur and iron–manganese. All recipes whose composition includes Fe-S or Fe-Mn, regardless of their denomination, are considered to be amber glass recipes. The others, with uranium and cadmium, are called yellow glass. The following section is dedicated to identifying and positioning the amber glasses within each arcanum.

#### 3.1.1. Marinha Grande Arcanum (MG)

Given the type of objects the factory produced, the MG arcanum is the one with the fewest amber glass recipes, a glass normally associated with utilitarian objects. Out of a total of 136 glass recipes, only 12 are of yellow/amber glass, 9% of the total. Considering that this is an 18th-century arcanum, most recipes are made of lead glass, as is the case for the other coloured recipes in the arcanum, and the chromophore is FeS, which, although not specified as an ingredient, was assumed to be the colouring agent in most recipes due to the presence of iron as an impurity in the sand, and the presence of sulphate compounds from the other ingredients. Some of the recipes even specify the origin of the sand (sometimes different from the other sand sources in other recipes), reinforcing the view that the iron comes from the sand and that it was a raw material known to the glassmakers who knew the characteristics of the sands from different locations and which one was suitable for producing this colour.

Some recipes’ names mention using this glass to produce beads. Unfortunately, with the information and context we have about the MG Factory and the arcanum today, almost all recipes have been defined as irreproducible, because although the basic recipe for producing amber glass is relatively simple, the most important information is the ratio and origin of the raw materials. Given that the industry has been studied emphasising political and social issues rather than technical ones, the lack of information on the raw materials used in Marinha Grande during this period makes it impossible to interpret most of the recipes.

#### 3.1.2. Castro and Oliveira Guerra Arcanum (COG)

The COG arcanum that belonged to two glassmakers, Castro and Oliveira Guerra, respectively, has a total of 161 glass recipes, 23 of which (14%) refer to yellow/amber glass. Most recipes have FeS or Fe-Mn as chromophore agents, and, like in the other arcana, the base composition is soda–lime silicate glass. The remaining recipes are associated with the manufacture of glass containing uranium, either alone or mixed with iron or copper. As we have seen, the glass made with uranium, according to these recipes, always contains potassium, alone or together with sodium. Again, there is no use of the word amber in these recipes, but instead the use of terms such as composition for French- or English-style bottles, gold or olive-oil coloured glass and topaz yellow in the Fe-Mn or FeS recipes and sulphur or canary colour in the recipes using uranium. Although all the recipes were considered legible, only eight were classified as reproducible. Like in other arcana, this is due to the use of cullet mixtures, or very specific cullet compositions, as for example, thick cullet, iron cullet, and green cullet. together with yellow cullet, among others. The basic composition is simple, but there are some variations in the type of reducing agent used (fir bark, corn flour, sulphur and coal were the most frequently mentioned), which may be explained by the fact that this arcanum accompanied the professional careers of both glassmakers who worked in various factories throughout time.

#### 3.1.3. Gaivotas Factory Arcanum (GF)

The arcanum with the greatest number of recipes is the GF arcanum, with 201 glass recipes, 15% of which are for producing amber/yellow glass. Most recipes use soda–lime glass as the base glass and FeS as the chromophore. Although the composition makes it easy to distinguish which recipes are for yellow glass or for amber glass, only one of the recipes is named as being for amber glass. The titles of all the other recipes mention yellow, champagne yellow, yellow for containers and China yellow (in the recipes with uranium). The recipes with cadmium or uranium, on the other hand, always have potassium in their vitreous matrix. Of the 31 recipes, only one is unreadable, but 15 are reproducible because most recipes include a cullet in the composition, indicating the use of dark and light cullet—a possible colour control technique—which makes it impossible to correctly interpret the recipe. The composition of most recipes is relatively simple, mentioning only silica, sodium, calcium, sulphur and flour. Recipes that include cullet also include flour and sulphur. It is worth noting that, except for two recipes that use charcoal, the most used reducing agent at GF was flour.

#### 3.1.4. Northern-Portuguese Glass Centre Arcanum (NPGC)

Regarding the arcanum belonging to the NPGC, it has a total of twenty-one glass recipes, and three are for amber glass, which corresponds to 14% of the recipes in total. All the recipes were classified as being Fe-Mn amber, although only two indicate both ingredients in their composition. The third recipe only mentions the use of manganese, but, as mentioned in the literature, manganese can only produce the amber colour in glass together with iron [6]. The iron in this recipe could, therefore, be an impurity derived from a raw material, namely from the sand. However, this recipe, like most of the recipes in this arcanum, mentions the use of quartz pebbles, which, according to the literature, have a low iron composition [21]. It is worth mentioning that this third recipe uses corn flour and sulphur as ingredients, which reinforces the view that iron is present in the composition and perhaps the manganese was just a routine addition, as it is an ingredient often used in glass production. The same recipe has a high percentage of cryolite (Na_3_AlF_6_), which could indicate that it is a yellow opal glass. All the recipes contain arsenic and zinc oxide, as a fining agent and a stabilizer, respectively. The names of the recipes are very basic and make no reference to what kind of objects could be produced with a particular glass composition.

**Figure 3:**
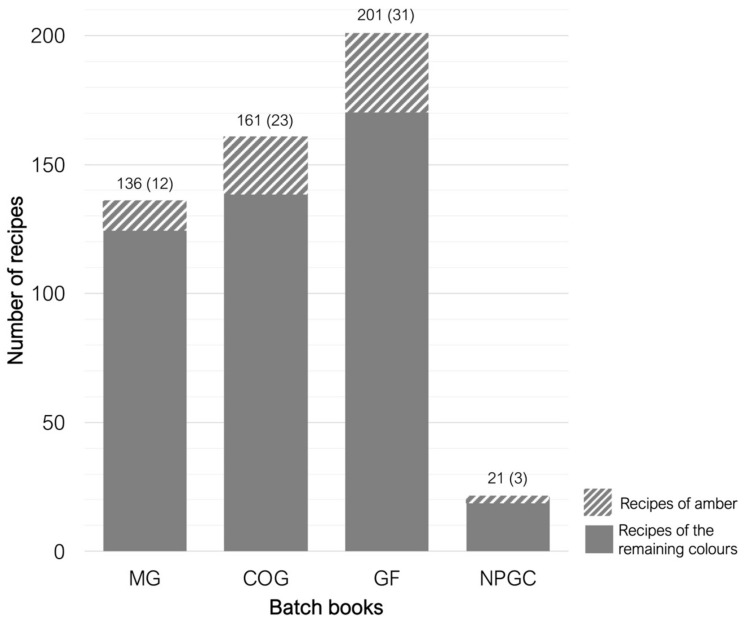
The total number of recipes in each arcanum. Diagonals indicate the number of yellow/amber recipes in each arcanum.

**Figure 4:**
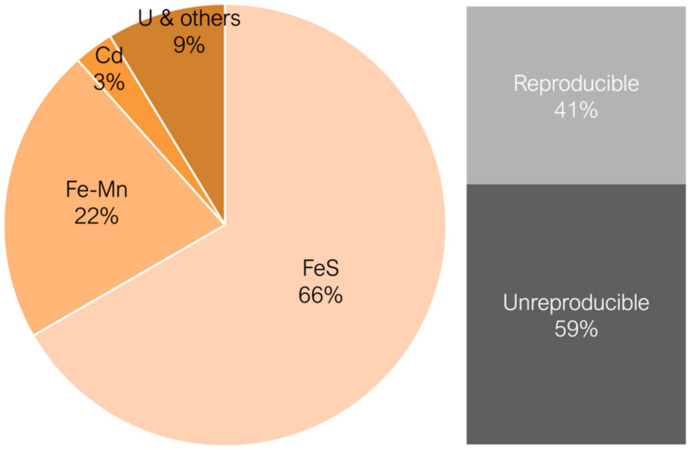
Different chromophores of glass recipes in the arcana and percentage of reproducible recipes for all arcana.

Although the four arcana belong to different chronologies and are different in length and number of recipes, the percentage of amber glass recipes is consistent and similar—9–15% (Figure 3). Most amber glass recipes are made from soda–lime glass, with iron or iron and manganese identified as chromophores (Figure 4). As mentioned earlier, iron alone does not produce the desired yellow/amber colour in the glass, but rather the chromophore FeS, which complexes in the glass matrix during the glass melting process. It should also be noted that most recipes are entitled as “recipe for yellow glass”, while just one uses clearly the word “amber” (in a recipe entitled “black glass (bottle amber)”). To better understand the interests of each factory, all recipes for yellow glass were considered, whether producing amber glass or not.

### 3.2. Recipe Reproduction

**Figure 5:**
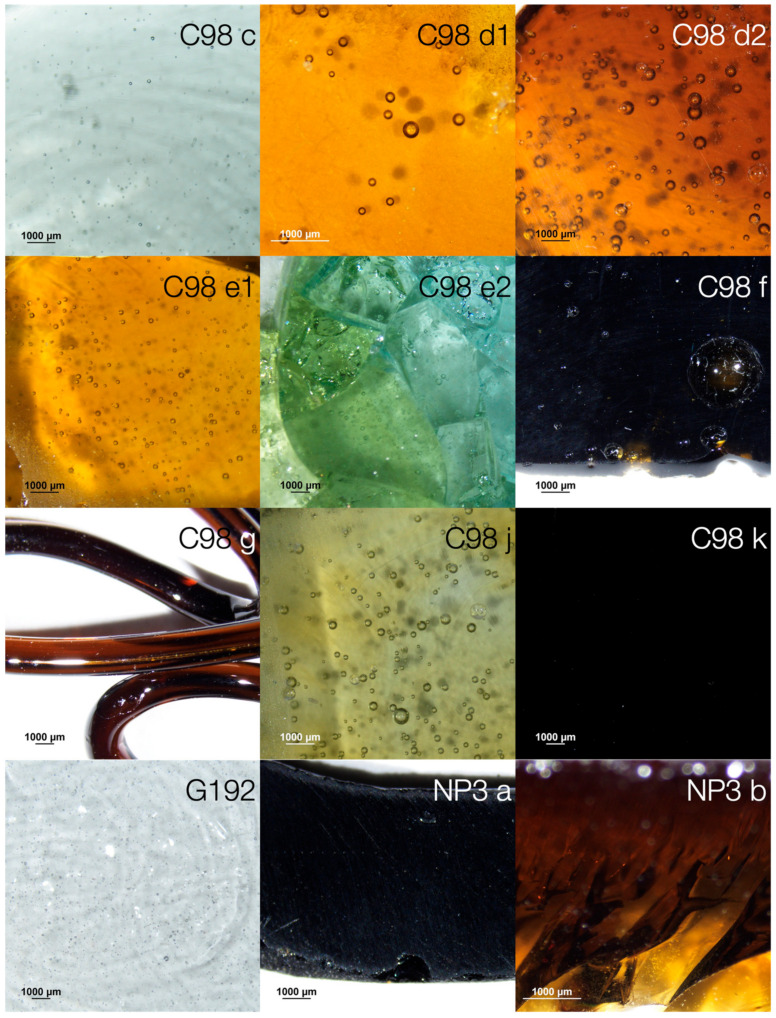
Pictures taken with the stereomicroscope of the samples produced.

From the five selected recipes, 21 attempts to reproduce amber glass were made and only 10 had a result between light and dark amber, as shown in Figure 5. It is worth noting that only two of the unsuccessful samples (because the result was either colourless or bluish colourless glass) have been considered in Figure 5 as examples, to make the data easier to read. Two versions of the NP3 recipe were reproduced, one of each of the GF recipes (G92, G130 and G192). The C98 appeared to be the simplest, with only four ingredients; for this reason, it was chosen to be explored in more depth, with more variables, and 15 versions were reproduced.

The two reproductions of the NP3 (recipe with Fe-Mn) resulted in a dark amber. The sample from the original recipe, **NP3 a**, produced a very dark, almost black glass. **NP3 b** had half the amount of Fe-Mn, which slightly altered the colour of the sample, but the difference is only visible in the finer areas.

Sample **G92** had elemental sulphur in its composition and flour as a reducing agent, but the result was a completely colourless glass. **G130** had charcoal as a reducing agent, but the same result was obtained. **G192**, despite having a more complex composition and more ingredients, resulted in the same as before, a colourless glass. In all three recipes, the problem is believed to be the amount of iron oxide added (0.057 wt.%), so for future work on amber glass from this factory, more information on its context is needed to ascertain the raw materials that were used.

Regarding recipe C98, it was chosen to be explored in more depth and with more variables. Sample **C98 a** used the amount of iron oxide from the analyses of the sand used by the Marinha Grande Factory (Barros 1969). As expected, due to the lack of a reducing agent, this recipe resulted in a colourless glass. In sample **C98 b**, more iron oxide was added, according to existing studies about the reproduction of amber glass [3]. Since there was still no reducing agent, the result was again a colourless glass. Therefore, in sample **C98 c,** charcoal was added to the composition. The result was a bluish colourless glass, as can be seen in Figure 5. According to other studies about natron composition, the problem seems to be the way sulphur is added [22]. The following results will therefore be presented together with the chemical characterisation of the glasses using WDXRF, to understand what may or may not be affecting the formation of the ferric sulphide complex.

### 3.3. Chemical Characterization

Although the original interpretation of the recipe did not produce the expected result, it provided an opportunity to explore and refine various factors. By successively adjusting several variables, we aimed to gain valuable insights into the role of each ingredient and to improve our understanding of the characteristics of the recipe. Table 4 shows the quantification of all the samples discussed in this section.

The authors are aware that some samples—C98 d2, C98 g and C98 j—present a higher content of alumina than expected. This is due to the alumina crucible contamination. Since the focus of the paper is on the colour control and variation, we believe that alumina does not play a role in this. However, it will have an impact on the mechanical properties of the glasses, which will be reflected in the Tg and Vickers Hardness values. When compared to historical samples, these will not have this influence from the crucible, as the extent of the contamination area of the crucibles used in the past (which were much bigger than the ones used in the current work) has already been studied [23].

Glass production in the 19th century already used chemical reagents produced in factories and laboratories, but their purity was not as rigorous and controlled as it is nowadays. For this reason, although the original recipe mentions the use of sodium carbonate, with the information we have today it is not possible to determine what impurities would be associated with it or even whether a specific mixture of sodium sources was used to produce amber. Therefore, based on these facts and the literature, the basic recipe was modified by changing the sodium source to a mixture of sodium bicarbonate, sodium sulphate and sodium chloride, in a ratio of 40:30:30 wt.% [3].

Sample **C98 d1** was the first successfully reproduced amber glass, with the following differences from the original: an increase in the amount of iron, the addition of sulphur with sodium and the addition of charcoal. From this recipe, two more samples were reproduced, **C98 d2** and **C98 d3**, based on the exact same recipe, with the aim to test the reproducibility. In the last one, the result was a colourless glass, despite having tried to reproduce it under the same conditions. This difference in result may have been due to an electrical fault during the melting process, or even differences in the percentage of water in the raw materials or batch moisture [4]. Looking at the characterization of the amber (**C98 d1** and **C98 d2**) and colourless (**C98 d3**) samples, the main difference is the SO_3_ value, which in the case of **C98 d3** is much higher than in the other samples. Samples **G92**, **G130** and **G192** also have a higher SO_3_ amount than samples **C98 d1** and **C98 d2**. According to the literature, the lower the amount of SO_3_, the more reduced the composition of the glass [4,6]. This shows that for the samples **G92**, **G130** and **G192,** the proportion, melting conditions or atmosphere in the glass matrix were not the most suitable for reducing the batch [4]. The differences in the percentage of SO_3_ in recipes of the same composition (for example, **C98 d1** and **C98 d3**) may indicate a greater volatilisation of SO_3_, which influences the redox conditions of the batch. Again, this may be due to batch moisture conditions.

As, according to the literature, the ideal proportion of iron oxide in amber glass is between 0 and 0.5 wt.%, the subsequent samples were made increasing this oxide from 0.05 wt.% to 0.2%. Again, three attempts were made with the same composition, and only **C98 e1** produced the amber colour. The **C98 e2** sample, although not completely colourless, has a much more subtle and heterogeneous colour. It is possible to see some areas which are more yellowish and others more bluish, possibly due to the higher concentration of Fe^3+^ and Fe^2+^ ions. The significant difference in characterization is again the SO_3_ amount, which in the case of sample **C98 e2** is below the detection limit, indicating that it has perhaps volatilised more than in the other samples. The difference in colour may have been due to a lack of homogenisation of the composition. Sample **C98 f** (30% charcoal) has the same amount of iron as **C98 e1** (20% charcoal) and different charcoal amounts, so a slight difference in colour was to be expected, but not too pronounced, as the amount of colourant is the same. However, the result for **C98 f** was an almost black glass, with the amber colour only visible in very thin areas, clearly due to the increased amount of reducing agent. The amount of reducing agent plays a fundamental role, sometimes even more important than the different amounts of iron. Samples **C98 d1** and **C98 e1**, with 0.1 and 0.2 wt.% iron oxide, respectively, and both with 20 wt.% charcoal, have a very similar visual result. However, samples **C98 e1** and **C98 f**, with the same amount of iron oxide (0.2 wt.%) and 20 and 30 wt.% charcoal, respectively, produced two completely different colours on the glass (amber, and a very dark amber resembling black, respectively).

Sample **C98 g** was made using the exact same recipe as sample **C98 e1**, but testing with the double of the total amount of raw materials. Instead of 60 g in total, 120 g was produced, and the result obtained was quite different for the two samples, with sample **C98 g** being noticeably darker. This shows that the result depends on several variables, including the batch quantity.

According to the literature, the ideal percentage of charcoal is between 10 and 20% [3]. Samples **C98 h** and **C98 i** contain 10% of charcoal and 10% and 20% of iron respectively. Both resulted in colourless glass, suggesting that although 10% of charcoal is sufficient to make amber glass according to the literature, it may not be enough to work with the proportions in this recipe.

Returning to 20% of charcoal, two more variations were tested. Sample **C98 j** has 0.05% iron oxide, an amount very similar to the G samples. The result was a very light, greenish amber, but it proved that it is possible to produce amber with extremely low iron content. Sample **C98 l** has 0.15% iron and although this is theoretically an amount that should produce an amber colour, since the samples with 0.10% and 0.20% (**C98 d1** and **C98 e1**) worked perfectly, the result was not satisfactory. Table 4 shows a higher SO_3_ value compared to the amber samples.

Finally, sample **C98 k** was an attempt to understand the influence of the base composition on Fe-Mn amber, starting from the C98 recipe, but with the same Fe-Si and Fe-Mn ratio as the **NP3 a** recipe. Despite the different base composition, the result was a glass that is nearly black, and indistinguishable from the **NP3 a** sample.

### 3.4. Thermal Properties

Thermal characterisation was carried out using DSC and dilatometry techniques (Table 5), which allowed for the measurement of the glass transition temperature (Tg), the dilatometric softening point (Td) and the thermal expansion coefficient (αL) of seven of the samples.

The glass transition temperatures of the samples measured by dilatometry range from 545.9 °C to 623.3 °C, where the samples coloured with FeS have a higher Tg, of around 600 °C, and the samples coloured with Fe-Mn have a lower Tg, of around 550 °C. It is worth noting that sample **C98 k** has the lowest Tg compared to **NP3 a** and **NP3 d**. This could be caused by the fact that the first sample has a higher sodium content, which has a direct impact on Tg. The lower Tg of NP3 a and NP3 b glasses have the advantage of requiring lower energy/temperature to melt the glass, making the process faster and less expensive.

As for the softening temperature, it gives us valuable information, and can even justify some choices about the uses of amber glass, mainly used for containers rather than for elaborate pieces. The Td of the samples varies between 583.6 °C and 663.6 °C. These temperatures are considered quite high, especially when compared to other compositions known to have been used for blown glass [9]. This characteristic possibly conditioned the type of object that could be produced with these compositions, because it needs a higher temperature for work that requires being constantly reheated to allow it to be blown.

With regard to αL, its values are between 7.0 and 12.2, which indicates that, despite the different compositions, these values remain similar. Samples with a lower αL have a higher percentage of Al_2_O_3_, since the presence of metal oxides like this means that there are fewer non-bridging oxygens available in the composition, making these glasses more resistant to expansion differences [24].

### 3.5. Vickers Hardness

The hardness tests were carried out to understand the properties of the produced historical glasses. By carrying out hardness analyses on samples produced from historical recipes and creating a database of their characterisation, when a reproduction is attributed to a historical object it is possible to hypothesize the thermal and mechanical properties of that object, without damaging it, in a kind of reverse engineering.

Nine samples were then measured to obtain the Vickers hardness, as shown in Table 6.

The hardness of most of the samples ranges between 565 HV0.5 and 655 HV0.5, values that are significantly high compared to other studies based on historical recipes [9], and even similar to the hardness of borosilicate glass [25].

The samples with the highest hardness are **C98 j**, **C98 k** and **NP3 b**, and the samples with the lowest hardness are **C98 d1**, **C98 e2** and **C98 f**. As can be seen in Figure 5, hardness does not seem to be connected to colour or colourants at all. However, by comparing their chemical characterisation in Table 4, we can see that the samples with the highest percentage of CaO and the lowest percentage of Al_2_O_3_ are the ones with the lowest hardness, coinciding with what is described in the literature [26].

### 3.6. Colour Characterisation

#### 3.6.1. Colorimetry

**Figure 6:**
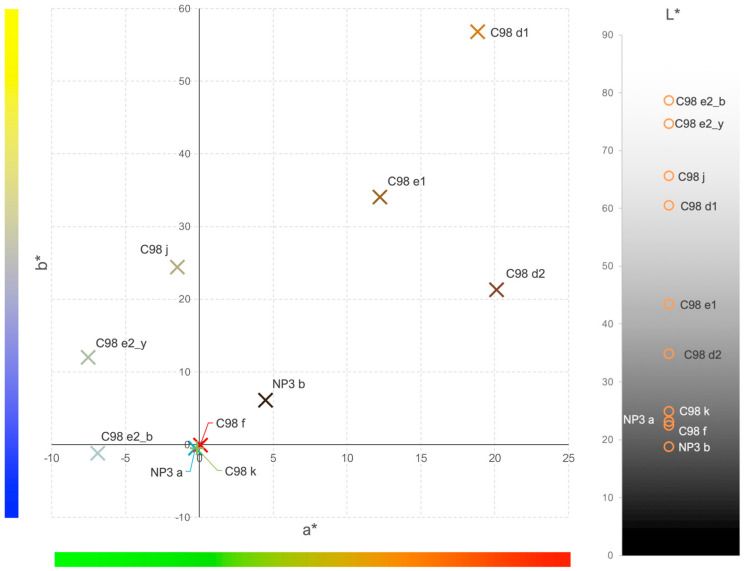
Scatter plot of the L*a*b* coordinates of the colorimetry measurements. Negative L* values closer to black and positive values closer to white; negative a* values closer to green and positive values closer to red; negative b* values closer to blue and positive values closer to yellow.

Since iron can give several colours to glass, such as blue, green and yellow, colour measurement is essential to understand the small differences in the colours of the various samples and which ones resemble each other despite the differences in composition. The scatter plot in Figure 6 represents the a* and b* coordinates of the CIE L*a*b* system.

Figure 6 shows that sample **C98 d1** is the closest to the yellow and red colours and sample **C98 d2** is much closer to the blue, despite their same composition. According to the literature, the most similar sample to the amber colour, in terms of a* and b* values, is sample **C98 e1** [27]. In the **C98 e2_b** and **C98 e2_y** samples (measurements taken in the bluest and yellowest areas of the sample C98 e2) it is noticeable that, although the colour change is not so significant, the analysis indicated that **C98 e2_y** has a higher b* value and is more on the yellow side of the plot. Point **C98 e3_b**, on the other hand, has negative values, and is closer to the blue area of the plot. In relation to sample **NP3 b,** it is visually quite dark, but the measurement clearly shows that its colour is much lighter than that of sample **NP3 a**. Samples **NP3 a**, **C98 f** and **C98 k**, as seen from Figure 6, are the darkest, almost black, so the b* and a* values are very close to zero. The L* colourimetric measurement confirms this, as samples C98 k, C98 f, NP3 a and NP3 b are the ones with the highest colour intensity.

#### 3.6.2. UV–Visible Absorbance Spectroscopy

**Figure 7:**
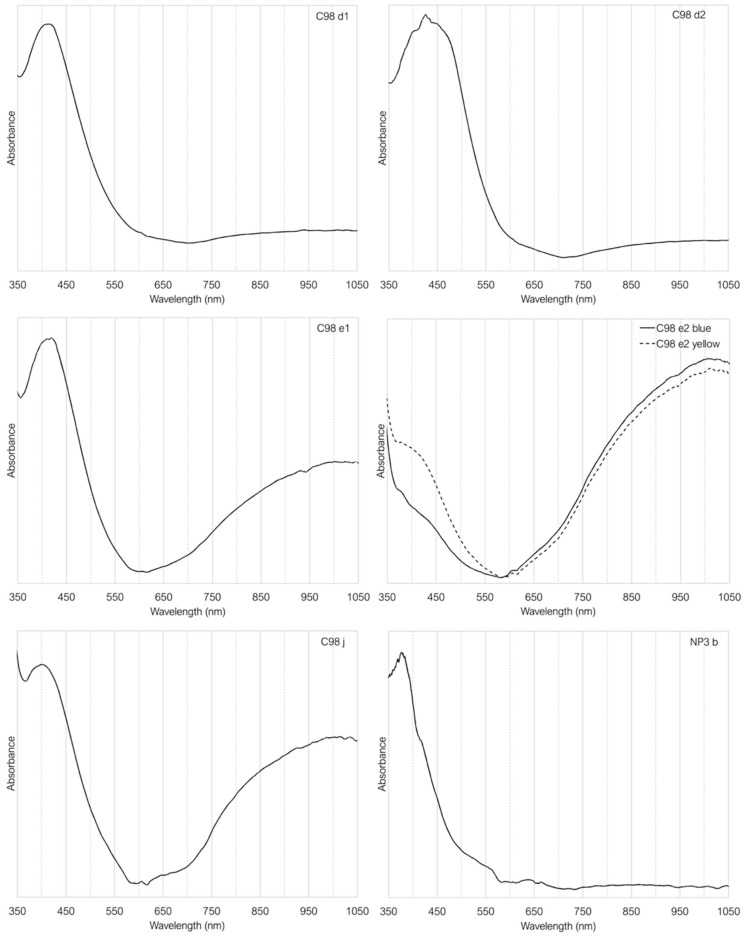
Absortion spectra of the amber glasses.

The chromophores were characterised using UV–Vis absorbance spectroscopy in six of the samples shown in Figure 7. The rest of the amber samples were not characterised by this technique because of their dark colour, which saturates the signal.

Samples **C98 d1** and **C98 d2,** as expected, show an absorption band around 425 nm, characteristic of the FeO_3_S sulphide ion. Additionally, a slight increase in absorption from 650 nm onwards is observed, indicating the possible presence of Fe^2+^ ions. Similarly, samples **C98 e1** and **C98 j** also exhibit a band at 425 nm; however, their spectra appear more absorbent beyond 650 nm, further suggesting again the presence of Fe^2+^ ions. Their characteristic bands are found at 1100 and 2100 nm, beyond the detection limit of the equipment used, but the broad absorption band presented between 650 and 1050 nm indicates its presence. The existence of Fe^2+^ ions (responsible for the blue colour) and Fe^3+^ ions (responsible for the yellow colour) produces the green colour in glass, one of the most common and easiest colours to produce, due to the natural presence of both iron oxidation states in glass [2]. Looking at Figure 6, it is possible to see that the colorimetry of the samples **C98 e1** and **C98 j** is shifted towards the green side of the graph, which may help to confirm the view that both oxidation states are present in these samples, although they were visually classified as amber glasses. In sample **NP3 b**, the Fe-Mn amber, the absence of Fe2+ ions are noticeable, indicating complete equilibrium between iron and manganese, as indicated by the equation (Equation (1)).

As mentioned above, the **C98 e2** sample is not homogeneous, so the analysis was carried out in two different areas, to see if this change was noticeable (Figure 7). It became apparent that the **C98 e2 yellow** point has a much more intense band at 425 nm from the Fe^3+^, while the Fe^2+^ region is slightly less intense. The **C98 e2 blue** point is exactly the opposite, as it is the bluest sample. It has less absorbance in the Fe^3+^ region and slightly more in the Fe^2+^ region.

## 4. Conclusions

In conclusion, this paper has explored the complex amber-glass colour technology from the 18th to 20th centuries, using historical recipes as primary sources. The research began by decoding and interpreting amber glass recipes from four Portuguese arcana, focusing on those with FeS or Fe-Mn as chromophores. These recipes were then reproduced in the laboratory and characterized chemically, thermally, and mechanically. The study revealed that producing FeS amber glass is highly intricate, with numerous variables that must be carefully controlled throughout the process. One recipe was selected for deeper analysis, and underwent significant adjustments as regards its interpretation. This was necessary because when converting historical recipes into modern laboratory protocols, it can be challenging to determine whether a recipe is ineffective or simply misinterpreted. By modifying the method of adding certain ingredients—while keeping the same ratio of elements as the original—we were able to explore whether specific raw materials were used in alternative forms that had not been initially considered.

This study introduced the critical observation that deeper knowledge of glassmaking processes does not always guarantee better results. Despite the widespread use of amber glass throughout history, it remains a challenge to reproduce it today with precise control over raw materials. Our results show that successful experimental results do not necessarily equate to a full understanding of all the variables involved, leading to inconsistencies in reproducing results. This adds a layer of complexity to the study of historical glass recipes and underlines the importance of not only interpreting historical methods, but also recognising the limitations that remain, even with modern advances.

As a result of this investigation, the creation of systematic guidelines for reproducing historical glass is proposed. These guidelines can help future studies on glass from different centuries, ensuring that new knowledge can continuously refine the process. By comparing reproduced glass with historical objects and sources, we can assess whether the information aligns or contradicts existing interpretations, which is essential to understanding the technological capabilities of the Portuguese glass industry during the period under consideration.

In addition to this impact, it is important to highlight information derived from the experiments that were carried out:

- Producing FeS amber glass proved far more complex than Fe-Mn amber. While Fe-Mn chromophores were easier to work with, they resulted in very dark colours, making it difficult to achieve softer, lighter amber colours.
- The percentage of SO_3_ and its volatility or complexation is critical. WDXRF analysis showed that samples with higher SO_3_ content tended to yield colourless glass. The method of sulphur addition also proved significant, with sulphur acting as expected, and producing the amber colour only when combined with sodium.
- Samples coloured with Fe-Mn (NP3 a and b) exhibited the lowest Tg, though all samples had high softening points.
- Vickers hardness tests showed that the samples were quite hard, with values approaching those of borosilicate glass. However, samples with higher calcium oxide and lower alumina oxide content were slightly less hard.
- Colorimetry results indicated that most amber samples skewed toward yellow and red, which is consistent with desired amber hues. While FeO_3_S is responsible for amber coloration, some UV–Vis spectra suggested the presence of Fe²⁺.

By uncovering the secrets of Portuguese glass factories through the study of batch books and recipes, this research bridges the past and present, offering critical insights into the craft behind the historic objects in our museums. Understanding these recipes not only improves the conservation and interpretation of these objects, but also contributes to the wider field of glass conservation. In this way, we ensure that the knowledge and techniques of past glassmakers are not lost to time, but continue to inform and enrich the future of glassmaking and conservation.

## Figures and Tables

**Table 1:** Transcription of the recipes selected for reproduction in kilograms. Northern Portuguese Glass Centre (NP), Castro and Oliveira Guerra glassmakers (C) and Gaivotas Factory (G).

NP3		C98		G92	
Pebbles	100	SiO^3^ S	200–100	Sand	30
Sand	-	NaOCO^2^	100–44	Lime	3.60
Feldspar	60	CaOCO^2^	72–30	Soda	15
Borax	26	Sulphur	2.8–1.4	Flour	1.5
Soda	12			Sulphur	0.200
Lime	2				
Dolomite	8	**G192**			
Boric acid	6	Sand	287.500		
Barite	-	Soda	113.410	**G130**	
Zinc oxide	4	Lime	34.000	Sand	150
Nitrate	4	Borax	1.138	Soda	66
Manganese	11	Sulphur	0.275	Lime	18
Iron oxide	13	Flour	1.900	Sulphur	0.200
Fluor-spar	1	Boric acid	3.980	Charcoal	0.950
Arsenic	0.300	NaCl	3.300	Boric acid	2

**Table 2:** Selected recipes and variations.

ID Recipe	Version	Main Recipe	Fe/Mn and Reducing Compounds (wt.%)	Reference
NP3	a	Original recipe	Original amount Fe-Mn	-
	b	Original recipe	Half amount of Fe-Mn	-
G92		Original recipe	0.057% Fe	Historical objects
G130		Original recipe	0.057% Fe	Historical objects
G192		Original recipe	0.057% Fe	Historical objects
C98	a	Original recipe	0.008% Fe	[17]
	b	Original recipe	0.2% Fe	[3]
	c	Original recipe	0.2% Fe + 10% charcoal	[3]
	d1	Natron as sulphur source	0.1% Fe + 20% charcoal	[3]
	d2	=	=	[3]
	d3	=	=	[3]
	e1	Natron as sulphur source	0.2% Fe + 20% charcoal	[3]
	e2	=	=	[3]
	e3	=	=	[3]
	f	=	0.2% Fe + 30% charcoal	[3]
	g	Double amount of e1	0.2% Fe + 20% charcoal	[3]
	h	Natron as sulphur source	0.2% Fe + 10% charcoal	[3]
	i	Natron as sulphur source	0.1% Fe + 10% charcoal	[3]
	j	Natron as sulphur source	0.05% Fe + 20% charcoal	[3]
	k	Original recipe	Fe-Mn from NP3a	-
	l	Natron as sulphur source	0.15% Fe + 20% charcoal	[3]

**Table 3:** Weighted quantities in grams.

	CaCO_3_	CaMg(CO_3_)_2_	Charcoal	Fe_2_O_3_	Flour	H_3_BO_3_	MnO_2_	Na_2_[B_4_O_5_(OH)_4_]·8H_2_O	Na_2_CO_3_	Na_2_SO_4_	NaAlS_i3_O_8_	NaCl	NaHCO_3_	NaNO_3_	SiO_2_	S	ZnO
NP3 a	0.403	1.623		2.641		1.218	2.237	5.284	2.436		12.197			0.816	20.327		0.811
NP3 b	0.41	1.625		1.1329		1.225	1.112	5.287	2.443		12.193			0.816	20.329		0.815
G92	3.578			0.022	1.495				14.909						29.834	0.199	
G130	3.794		0.202	0.017		0.421			13.935						31.604	0.043	
G192	3.815			0.023	0.213	0.449		0.132	12.726			0.374			32.252	0.033	
C98 a	9.615			0.002					13.348						26.678	0.374	
C98 b	9.603			0.101					13.343						26.679	0.374	
C98 c	9.605		5.014	0.1					13.344						26.684	0.376	
C98 d1	10.348		10	0.05						4.765		4.765	6.354		23.767		
C98 d2	8.874		10.002	0.052						4.943		4.942	6.59		24.654		
C98d3	8.878		10.026	0.051						4.938		4.939	6.587		24.65		
C98 e1	10.348		10	0.1						4.765		4.765	6.354		23.767		
C98 e2	8.875		10.005	0.102						4.941		4.941	6.592		24.651		
C98 e3	8.876		10.008	0.1						4.943		4.941	6.59		24.663		
C98 f	10.348		15	0.1						4.765		4.765	6.354		23.767		
C98 g	20.696		20	0.2						9.53		9.53	12.708		47.534		
C98 h1																	
C98 h2	8.875		5.003	0.105						4.943		4.945	6.593		24.657		
C98 i	8.874		5.001	0.049						4.941		4.943	6.592		24.653		
C98 j	8.873		10.006	0.027						4.945		4.945	6.593		24.656		
C98 k	9.605			3.468			2.934		13.339						26.673		
C98 l	8.877		10.004	0.076						4.944		4.943	6.591		24.653		

Letters: identification of the different versions of the original recipe. Numbers: identification of the several reproductions of the same recipe.

**Table 4:** Wavelength-dispersive X-ray fluorescence spectroscopy (WDXRF) results in weight percentage of oxides.

SAMPLE	Sum Before Normalization	Na_2_O	MgO	Al_2_O_3_	SiO_2_	SO_3_	Cl	K_2_O	CaO	MnO	Fe_2_O_3_	ZnO
NP3 a	80.1	8.07	0.95	11.0	64.5	-	-	0.20	2.11	4.27	6.85	1.89
NP3 b	74.3	8.24	0.89	13.0	67	-	-	0.21	2.34	2.25	3.92	2
G92	73.5	18.4	-	0.8	73.9	0.91	-	0.05	5.83	-	0.07	0.01
G130	69.7	16.5	-	0.2	76.5	0.18	-	0.06	6.44	-	0.06	0.01
G192	74.4	16.3	-	0.9	76.1	0.1	0.48	0.05	6.05	-	0.06	-
C98 a	81.2	16.8	-	0.6	66.4	0.85	-	0.03	15.2	-	-	-
C98 b	81.1	16.9	0.27	1.27	65.3	0.50	-	0.02	15.4	-	0.32	-
C98 c	78.2	15.3	-	1.4	66.1	0.76	-	0.13	16	-	0.27	-
C98 d1	84.4	11.1	0.19	2.24	66.6	0.08	0.29	0.12	19.1	-	0.18	0.01
C98 d2	80.9	9.96	0.14	6.45	66.4	0.08	0.18	0.09	16.5	-	0.18	0.01
C98 d3	80.4	8.54	0.22	0.84	70	0.23	0.51	0.11	19.3	-	0.19	-
C98 e1	58.4	9.91	0.2	1.88	66.3	0.05	0.32	0.13	20.8	-	0.41	0.01
C98 e2	76.3	11.4	0.18	2.16	68.2	-	0.22	0.13	17.3	-	0.36	0.01
C98 e3	74.3	9.99	-	0.63	69.9	0.24	0.56	0.12	18.1	-	0.37	-
C98 f	68.2	10.1	0.18	2.75	64.6	1.07	0.63	0.19	20.1	-	0.37	0.01
C98 g	82.5	8.62	0.19	5.17	67.5	0.37	0.94	0.44	16	-	0.64	0.01
C98 h	86	11.8	-	1.28	69.9	0.31	0.57	0.05	15.7	-	0.34	-
C98 i	71.5	9.81	-	3.24	67.6	0.17	0.4	0.09	18.5	-	0.19	0.01
C98 j	82.7	10.4	0.14	4.55	67.8	-	0.18	0.12	16.6	-	0.11	0.01
C98 k	81.1	13.1	-	3.57	54.4	-	-	0.08	13.5	5.8	9.47	0.02
C98 l	83.3	10	-	1.71	69.1	0.3	0.36	0.12	18.1	-	0.22	0.01

Letters: identification of the different versions of the original recipe. Numbers: identification of the several reproductions of the same recipe.

**Table 5:** Results of the thermal analyses.

	C98 d1	C98 d2	C98 e1	C98 f	C98 k	NP3 a	NP3 b
T_g (onset)_ DSC (°C)	608.9	617.4	607.3	-	-	582.9	583.0
T_g (onset)_ DIL (°C)	608.7	623.3	611.7	605.1	545.9	580.7	593.4
Td (°C)	642.9	663.6	649.3	650	583.6	643.9	659
αL (50–500 °C) × 10^−6^ (°C^−1^)	9.7	9.6	9.5	10.6	12.2	7.1	7.0

**Table 6:** Vickers hardness of the amber samples.

	C98 d1	C98 d2	C98 e1	C98 e2	C98 j	C98 j	C98 k	NP3 a	NP3 b
HV 0.5	565	574	590	574	627	567	605	568	655
STDEV	19.8	8.8	15.2	4.4	15.4	8.1	22.7	14.5	16.2
Mean percentage	3.51%	1.53%	2.63%	0.77%	2.46%	1.41%	3.75%	2.55%	2.48%

## Data Availability

The original contributions presented in the study are included in the article, further inquiries can be directed to the corresponding authors.
